# Venous Thrombosis within 30 Days after Vaccination against SARS-CoV-2 in a Multinational Venous Thromboembolism Registry

**DOI:** 10.3390/v14020178

**Published:** 2022-01-18

**Authors:** Behnood Bikdeli, David Jiménez, Pablo Demelo-Rodriguez, Francisco Galeano-Valle, José Antonio Porras, Raquel Barba, Cihan Ay, Radovan Malý, Andrei Braester, Egidio Imbalzano, Vladimir Rosa, Ramón Lecumberri, Carmine Siniscalchi, Ángeles Fidalgo, Salvador Ortiz, Manuel Monreal, for the RIETE Investigators

**Affiliations:** 1 Cardiovascular Medicine Division, Brigham and Women’s Hospital, Harvard Medical School, Boston, MA 02115, USA; 2 Center for Outcomes Research and Evaluation (CORE), Yale School of Medicine, New Haven, CT 06510, USA; 3 Clinical Trials Center, Cardiovascular Research Foundation, New York, NY 10019, USA; 4 Respiratory Department, Hospital Ramón y Cajal and Universidad de Alcalá (IRYCIS), 28034 Madrid, Spain; djimenez.hrc@gmail.com; 5 Centro de Investigación Biomédica en Red de Enfermedades Respiratorias (CIBERES), 28029 Madrid, Spain; 6 Department of Internal Medicine, Hospital General Universitario Gregorio Marañón, 28007 Madrid, Spain; pbdemelo@hotmail.com (P.D.-R.); paco.galeano.valle@gmail.com (F.G.-V.); 7 Instituto de investigación sanitaria Gregorio Marañón (IiSGM), 28007 Madrid, Spain; 8 School of Medicine, Universidad Complutense de Madrid, 28040 Madrid, Spain; 9 Department of Internal Medicine, Hospital Universitario Joan XXIII de Tarragona, 43005 Tarragona, Spain; aporras.hj23.ics@gencat.cat; 10 Department of Internal Medicine, Hospital Rey Juan Carlos, 28933 Madrid, Spain; raquel.barba@hospitalreyjuancarlos.es; 11 Department of Medicine I - Clinical Division of Haematology and Haemostaseology, Medical University of Vienna, 1090 Vienna, Austria; cihan.ay@meduniwien.ac.at; 12 Department of Cardiovascular Medicine I, Faculty of Medicine, Charles University in Prague, University Hospital Hradec Kralove, 500 05 Hradec Kralove, Czech Republic; malyr@volny.cz; 13 Department of Haematology, Azrieli Faculty of Medicine, Bar-Ilan University, 13195 Safed, Israel; AndreiB@gmc.gov.il; 14 Department of Clinical and Experimental Medicine, A.O.U Policlinico “G. Martino”, 98124 Messina, Italy; eimbalzano@unime.it; 15 Department of Internal Medicine, Hospital Universitario Virgen de Arrixaca, 30120 Murcia, Spain; vladi_medico@yahoo.es; 16 Department of Haematology, Clínica Universidad de Navarra, 31008 Pamplona, Spain; rlecumber@unav.es; 17 Center for Biomedical Research Network on Cardiovascular Diseases (CIBERCV), Instituto de Salud Carlos III, 28029 Madrid, Spain; 18 Department of Angiology, Azienda Ospedaliera Universitaria, 43126 Parma, Italy; csiniscalchi@ao.pr.it; 19 Department of Internal Medicine, Hospital Universitario de Salamanca, 37007 Salamanca, Spain; angelesfidalgo@gmail.com; 20 Department of Statistics, Universidad Autónoma Madrid, 28049 Madrid, Spain; salvador.ortiz@shmedical.es; 21 S&H Medical Science Service, 28034 Madrid, Spain; 22 Faculty of Health Sciences, Universidad Católica San Antonio de Murcia (UCAM), 30107 Murcia, Spain; mmonreal.germanstrias@gencat.cat

**Keywords:** venous thromboembolism, COVID-19, SARS-CoV-2, vaccination

## Abstract

Background: Venous thromboembolism (VTE)—including deep vein thrombosis, pulmonary embolism, and cerebral venous sinus thrombosis (CVST)—may occur early after vaccination against the severe acute respiratory syndrome coronavirus 2 (SARS-CoV-2). We sought to describe the site, clinical characteristics, and outcomes of VTE after vaccination against SARS-CoV-2. Methods: In a prospective study using the Registro Informatizado de Enfermedad TromboEmbólica (RIETE) platform, patients with VTE 4–30 days after vaccination against SARS-CoV-2 (1 February 2021 through 30 April 2021) were included. VTE patients recruited from the same centers into RIETE in the same months in 2018–2019 were selected as the reference group. All-cause mortality and major bleeding were the main study outcomes. Results: As of 30 April 2020, 102 patients with post-vaccination VTEs had been identified (28 after adenovirus-based vaccination [ChAdOx1 nCov-19; AstraZeneca] and 74 after mRNA-based vaccination [mRNA-1273; Moderna, and BNT162b2; Pfizer]). Compared with 911 historical controls, patients with VTE after adenovirus-based vaccination more frequently had CVST (10.7% vs. 0.4%, *p* < 0.001) or thrombosis at multiple sites (17.9% vs. 1.3%, *p* < 0.001), more frequently had thrombocytopenia (40.7% vs. 14.7%, *p* < 0.001), and had higher 14-day mortality (14.3% vs. 0.7%; odds ratio [OR]: 25.1; 95% confidence interval [CI]: 6.7–94.9) and major bleeding rates (10.3% vs. 1.0%, OR: 12.03, 95% CI: 3.07–47.13). The site of thrombosis, accompanying thrombocytopenia, and 14-day mortality rates were not significantly different for patients with VTE after mRNA-based vaccination, compared with historical controls. Conclusions: Compared with historical controls, VTE after adenovirus-based vaccination against SARS-CoV-2 is accompanied by thrombocytopenia, occurs in unusual sites, and is associated with worse clinical outcomes.

## 1. Introduction

COVID-19 has resulted in substantial mortality and morbidity around the world [1]. Respiratory distress and thrombotic events-particularly venous thromboembolism (VTE)—are among the major concern in these patients [2,3,4,5]. The availability of several effective vaccines has been a key step toward reducing the burden of COVID-19 [6,7,8,9].

There have been, however, recent reports of VTE early after vaccination for severe acute respiratory syndrome coronavirus-2 (SARS-CoV-2), particularly with adenovirus-based vaccines (including ChAdOx1 nCov-19 [AstraZeneca] and Ad26.COV2.S [Johnson & Johnson/Janssen]) [10,11,12,13,14,15,16,17,18]. In the existing reports, some of the cases of thrombosis occurred in unusual sites such as cerebral venous sinus thrombosis (CVST) and splanchnic vein thrombosis ([SVT], including portal, hepatic, mesenteric, renal, and gonadal veins). These events were reported mostly in women of childbearing age, associated with thrombocytopenia and antibodies against platelet factor-4 (PF-4), referred to as vaccine-induced thrombotic thrombocytopenia (VITT) [10,11,12,13,14,15,19]. This issue has caused concern among the public, clinicians, and policy-makers, with restrictions or pauses being implemented for the use of adenovirus-based SARS-CoV-2 vaccines [11,20]. It remains uncertain whether the majority of thrombotic events in the early post-vaccination period are associated with thrombocytopenia and PF-4 antibodies. In addition, the existing reports are mostly from small series, with limited ability to understand the patient characteristics and clinical outcomes, compared with patients with VTE not associated with recent vaccination.

Using the platform of a multinational ongoing VTE registry, this study was designed to prospectively collect information related to VTE between 4–30 days after vaccination for SARS-CoV-2. We report the events according to the site of thrombosis and type of vaccine and provide the time course of VTE after vaccination, clinical characteristics, and outcomes of patients with VTE from the same enrolling centers in similar periods of prior years as reference.

## 2. Methods

### 2.1. Data Source

For this study, we used the platform of the Registro Informatizado de Enfermedad TromboEmbólica (RIETE) registry, a multinational registry of patients with VTE. This multinational registry nitially started in 2001, focusing on deep vein thrombosis (DVT) and pulmonary embolism (PE) in Spain; the registry subsequently expanded to several other countries and other forms of VTE including CVST and SVT. As of 30 April 2021, there are 91,568 cases of DVT or PE, 210 cases of CVST, 936 cases of SVT, and 536 cases of DVT in more than one territory in RIETE. Additional details about the methodology of RIETE have been described previously [21].

For the current non-interventional study, the RIETE platform of investigators, data entry system, and coordinating center were used for prospective enrollment of patients with VTE after vaccination for SARS-CoV-2. Patients or their healthcare proxies provided informed consent in accordance with the Ethics Committee at each hospital.

### 2.2. Patients

From 1 February 2021, through 30 April 2021, patients with imaging-confirmed diagnosis of VTE between 4–30 days after vaccination for SARS-CoV-2 were included in this study. To provide a source of reference for patient characteristics, treatment pattern, and outcomes, patients from RIETE who were enrolled between 1 February 2018 through 30 April 2018, and 1 February 2019 through 30 April 2019, from the same centers that included post-vaccination cases of VTE, were selected.

### 2.3. Exposure Variable and Other Variables of Interest

The main exposure variable of interest was administration of a SARS-CoV-2 vaccine dose within 4–30 days prior to VTE. The type of vaccine and the dose (first or second) was also determined. The site of thrombosis was grouped into four categories: (a) DVT in the lower or upper extremities or PE; (b) CVST; (c) SVT; and (d) thrombosis in multiple sites defined as at least two of a, b, and c. Other variables include VTE risk factors and presenting clinical features. Among laboratory tests, thrombocytopenia (defined as platelet count < 150,000/μL) was assessed at baseline. In those with thrombocytopenia, testing status for PF-4 antibody and test results (if tested) were reported, as were additional laboratory markers such as fibrinogen and D-dimer.

### 2.4. Outcomes

The main study outcome was all-cause mortality at 14 days since diagnosis of VTE. Major bleeding events were also ascertained. Bleeding was assessed according to RIETE criteria and considered as major if the events required ≥ 2 units of blood transfusions, were overt, involved a critical area (retroperitoneal, spinal, intracranial, or intrapericardial), or were fatal. This definition closely approximates that of the International Society on Thrombosis and Haemostasis for major bleeding [22].

### 2.5. Statistical Analysis

This study was designed for description of the clinical features and outcomes in this patient subset. No single hypothesis was pre-selected as primary. Although a traditional significance level of 0.05 was chosen for presentation purposes, *p*-values should be considered exploratory. To compare the clinical outcomes of patients with post-vaccination VTE versus controls from the same centers during 2018 and 2019, in addition to unadjusted analyses, mixed effects models with a random intercept for enrolling sites and odds ratio as the effect measure were used. Covariates for adjustment included age, sex, thrombocytopenia, cerebral or splanchnic vein involvement, cancer, and history of VTE. In all models, historical controls were the reference group. Time-to-event was shown with cumulative incidence curves, and hazard ratio was used for the effect measure for time-to-event analysis.

## 3. Results

**Figure 1:**
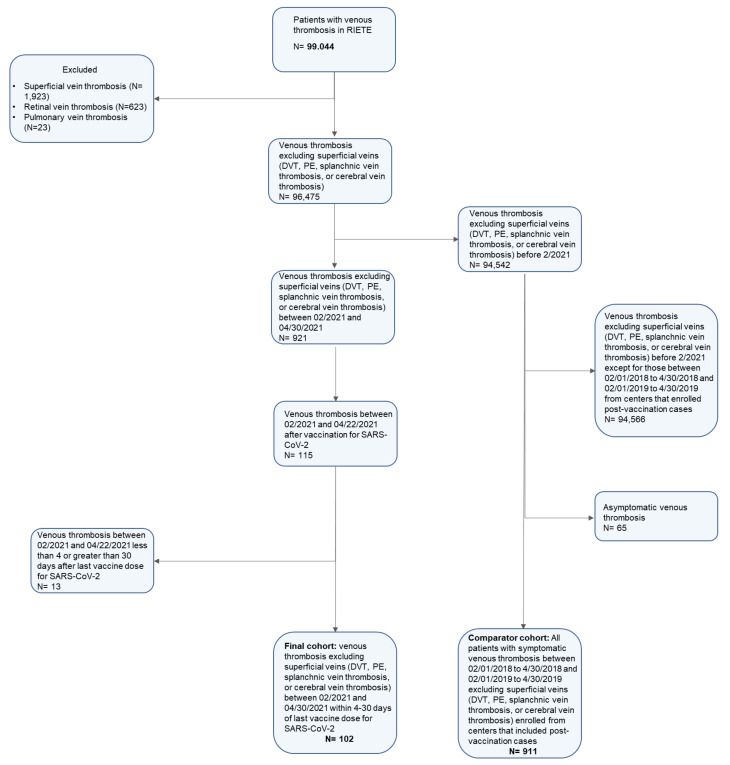
Study Flow Diagram. N: sample size.

Between 1 February 2021 and 30 April 2021, 115 patients with VTE after vaccination for SARS-CoV-2 were identified, of whom 102 occurred between 4–30 days post-vaccination and were included in the current study. Of these, 28 occurred after vaccination with adenovirus-base vaccines (28 after AstraZeneca) and 74 occurred after vaccination with mRNA-based vaccines (63 after Pfizer and 11 after Moderna vaccines). No patients with VTE after Johnson and Johnson vaccination were identified (Figure 1). All cases had acute symptomatic VTE. Table S1 summarizes the individual patient information for the study participants. In similar months from 2018 and 2019 and from the same enrolling centers, a total of 911 patients with symptomatic VTE were enrolled in RIETE.

### 3.1. VTE Presentation and Clinical Characteristics

Baseline characteristics of patients with VTE are summarized in Table 1. Mean age in patients with VTE after adenovirus-based vaccination, mRNA-based vaccination, and historical controls were 53.8 ± 2.5, 78.3 ± 1.7, and 65.5 ± 0.6 years, respectively. The proportion of patients with VTE who did not have any traditional major risk factors (active cancer, surgery or medical hospitalization within the prior 30 days, or pregnancy/puerperium status) was higher in those with VTE after adenovirus-based vaccination (85.7%) or mRNA-based vaccination (66.2%) compared with historical controls (*p* < 0.001, and *p* = 0.01, respectively). Patient characteristics according to individual vaccine types are summarized in Table S2.

Compared with 911 historical controls with symptomatic VTE from 2018 and 2019, patients with VTE after adenovirus-based vaccination against SARS-CoV-2, more frequently presented with CVST (10.7% vs. 0.4%, *p* < 0.001) or thrombosis at multiple sites (17.9% vs. 1.3%, *p* < 0.001), and more frequently had thrombocytopenia (platelet count < 150,000/fL) at baseline (40.7% vs. 14.7%, *p* < 0.001).

Compared with historical controls, patients with VTE after mRNA-based vaccination against SARS-CoV-2 did not have a significantly different rate of patients presenting with CVST (2.7% vs. 0.4%, *p* = 0.07) and thrombosis at multiple sites (1.4% vs. 1.3%, *p* > 0.99), or thrombocytopenia at baseline (13.5 vs. 14.7%, *p* = 0.78).

Among patients with VTE and thrombocytopenia after vaccination for SARS-CoV-2, PF-4 antibodies were tested in five patients with VTE after adenovirus-based vaccination and were positive in five patients. PF-4 antibodies were not tested in any patients with VTE after vaccination with mRNA-based vaccines. Table S3 summarizes the patients characteristics for those who had post-vaccination VTE with thrombocytopenia at baseline.

### 3.2. Initial Treatment

Most patients received initial treatment with anticoagulation. Use of fibrinolytic therapy, thrombectomy, and vena cava filters was rare in all groups. No patient with CSVT underwent endovascular thrombectomy. A summary of the initial treatment pattern based on the type of VTE is provided in Table 2.

### 3.3. Clinical Outcomes

Compared with historical controls, patients with VTE after adenovirus-based vaccination against SARS-CoV-2 had higher odds of mortality at 14 days (14.3% vs. 0.7%; odds ratio [OR]: 25.14, 95% confidence interval [CI]: 6.66–94.91). Results were consistent in multivariable analysis (OR: 13.24, 95% CI: 1.44–121.3). In addition, patients with VTE after adenovirus-based vaccination against SARS-CoV-2 had higher odds of major bleeding compared with historical controls in unadjusted (OR: 12.03, 95% CI: 3.07–47.13, *p* < 0.001) and multivariable adjusted analyses (OR: 9.03, 95% CI: 1.07–76.13, *p* = 0.043).

**Figure 2:**
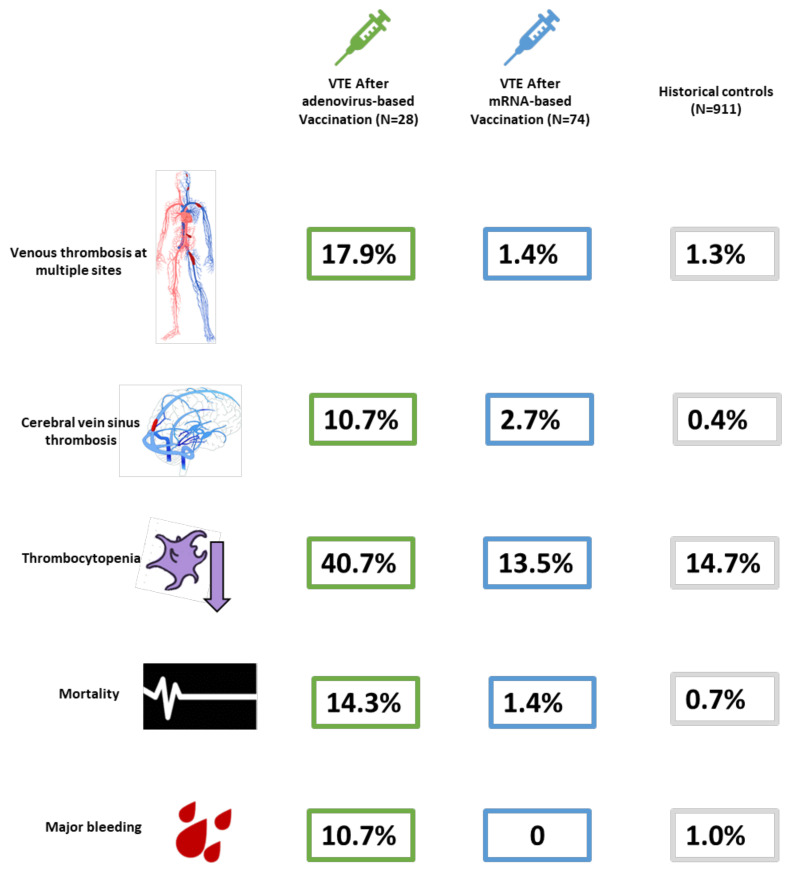
Main Features of Presentation and Outcomes in the Study Groups. N means sample size.

Patients with VTE after mRNA-based vaccination against SARS-CoV-2 did not have a significantly different rate of 14-day mortality compared with historical controls in unadjusted (OR: 2.07, 95% CI: 0.25–17.39, *p* = 0.504) or multivariable adjusted (OR: 1.05, 95% CI: 0.11–10.16, *p* = 0.964) analyses. None of the patients with VTE after receiving mRNA-based vaccination against SARS-CoV-2 suffered from major bleeding (Table 3, Figure 2).

**Figure 3:**
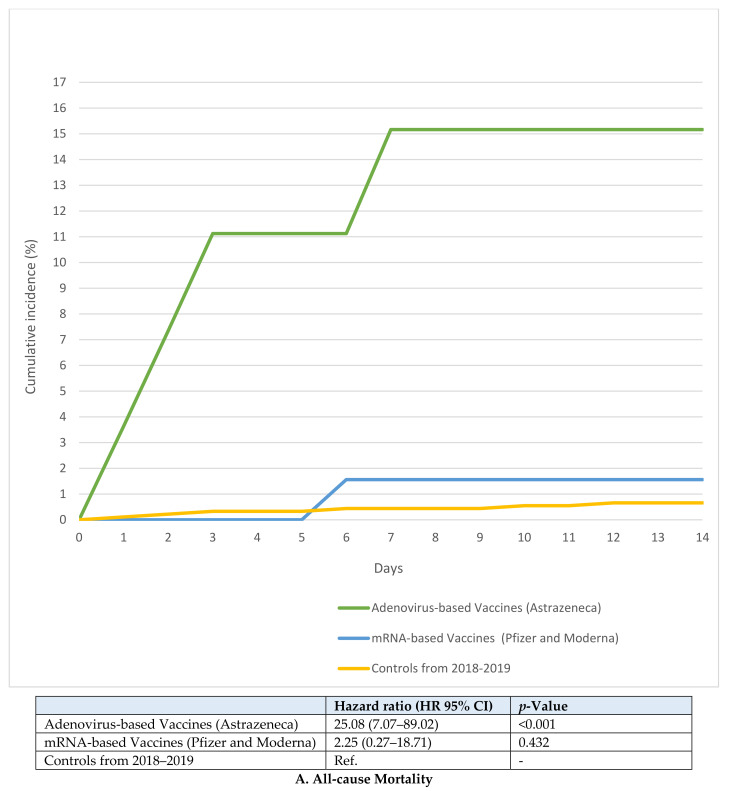

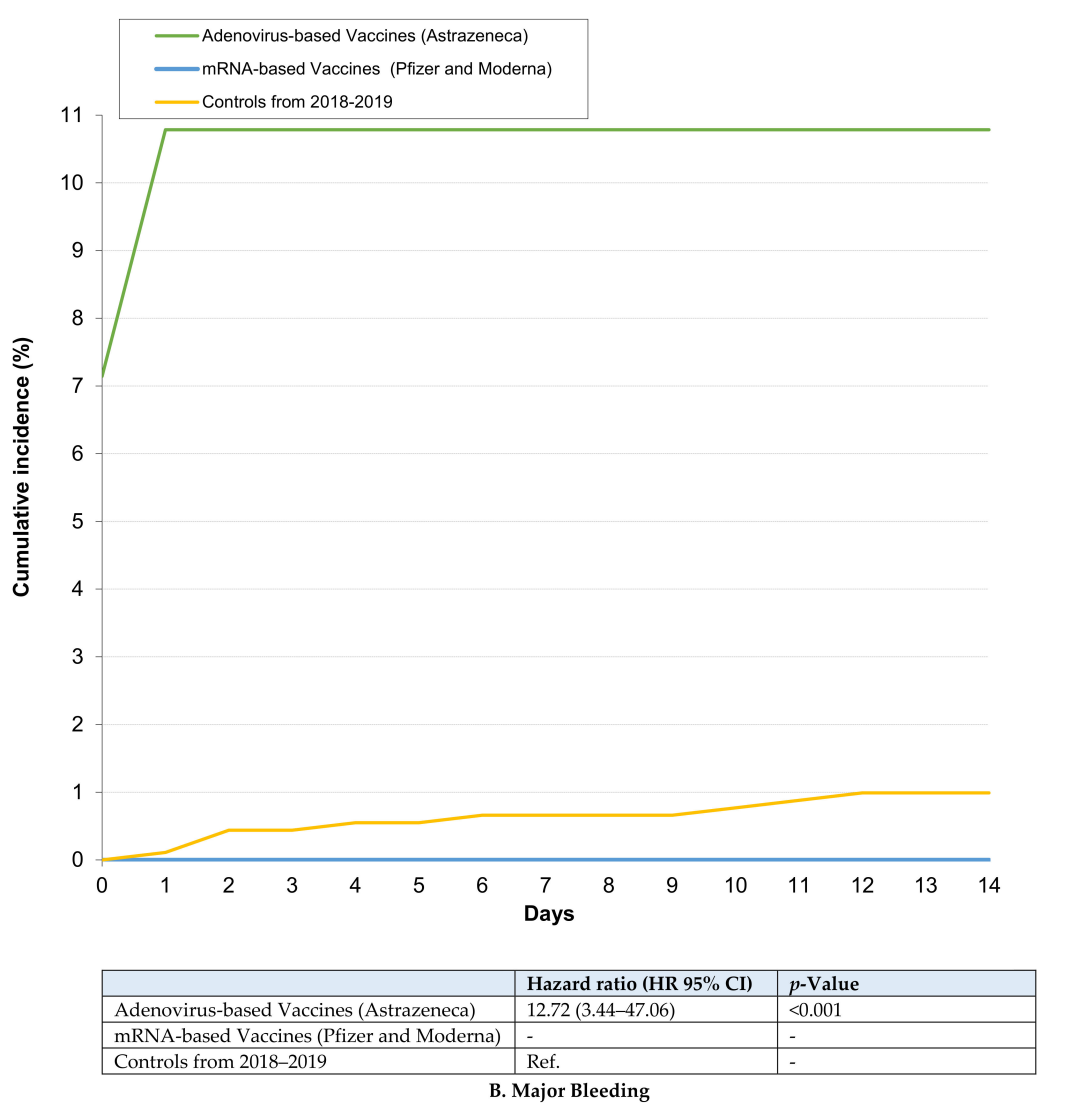
Cumulative incidences for all-cause mortality (**A**) and major bleeding (**B**) in patients with Post-SARS-CoV-2 vaccination VTE compared with patients with VTE in 2018 and 2019. – means not applicable.

Time-to-event analyses were similar. Patients with VTE after adenovirus-based vaccination against SARS-CoV-2 had a higher hazard for all-cause mortality compared with historical controls (hazard ratio [HR]: 25.08, 95% CI: 7.07–89.02, *p* < 0.001, Figure 3A). In contrast, patients with VTE after receiving mRNA-based vaccination did not have a higher hazard for all-cause mortality compared with historical controls (HR: 2.25, 95% CI: 0.27–18.71, *p* = 0.432). Findings were similar for time-to-event analysis for bleeding (Figure 3B).

## 4. Discussion

This report based on an international study found several key distinctions in presenting features and clinical outcomes in patients with VTE after vaccination against SARS-CoV2, compared with historical controls. VTE after vaccination against SARS-CoV2, both with adenovirus-based vaccination and mRNA-based vaccination, more frequently occurred in patients without traditional VTE risk factors, compared with controls. Thrombocytopenia at presentation was more frequently observed in patients with adenovirus-based vaccination but not those with mRNA-based vaccination, compared with historical controls. Patients with VTE after adenovirus-based vaccination but not those with mRNA-based vaccination, had a greater relative frequency of VTE at unusual sites, compared with historical controls. As such, VTE after adenovirus-based vaccination, but not mRNA-based vaccination, had higher rates of mortality and major bleeding, compared with historical controls.

This study extends the existing knowledge about VTE after vaccination against SARS-CoV-2 reported in prior studies [12,13,14,15,16,19,23]. Several recent reports suggested thrombocytopenia and VITT to be a unique feature of VTE events after adenovirus-based vaccination against SARS-CoV-2 [12,13,14,15,16]. Similar to those studies, we noted that 11 out of 27 patients with VTE after adenovirus-based vaccination for SARS-CoV-2 had thrombocytopenia. In those who were tested for PF-4, five out of five tested positive, with features consistent with VITT. In addition, we noted that 10 out of 28 patients had VTE in forms other than DVT or PE. The current study also indicates that the majority of VTE events after adenovirus-based vaccination against SARS-CoV-2 are not associated with thrombocytopenia and that such events, unlike thrombotic events occurring in unusual sites or associated with thrombocytopenia, have a relatively benign course.

This study provides information on a large series of patients with VTE in close proximity to mRNA-based vaccination against SARS-CoV-2. VTE events after mRNA-based vaccination less frequently occurred on the background of traditional VTE risk factors compared with historical controls. This finding was similar to VTE events after adenovirus-based vaccination. However, VTE events after mRNA-based vaccination, unlike VTE events after adenovirus-based vaccination, were not frequently associated with thrombocytopenia, and did not occur more frequently at unusual sites of thrombosis or have higher mortality rates, compared with historical controls.

This study has several limitations. First, RIETE is a registry of patients with VTE. Therefore, the study is unable to identify the attributable risk of different types of SARS-CoV-2 vaccines for incident VTE based on vaccination rates. Greater number of patients with VTE after mRNA-based vaccination should not be perceived as rates. The numbers observed in this study are likely related to the type of vaccines used in countries from participating enrolling centers. Further, by design, it does not capture patients who initially presented with arterial thrombotic events. However, the majority of cases of vaccine-associated thrombosis have been in the form of VTE. Second, similar to other registries, patient entry into RIETE is voluntary, although investigators are strongly encouraged to enter all their consecutive patients. For the current study, general as well as specific queries were sent to enrolling sites. Prior studies have shown excellent correlation of patient characteristics between RIETE enrollees and national administrative databases [24]. Third, this was a non-interventional study and some laboratory or clinical parameters (particularly PF-4 antibodies) were not collected by all treating clinicians. Reasons for such missing information include lack of global knowledge of VITT during the study period, since some of these events occurred early in the vaccination era before VITT was well described, lack of availability of PF-4 antibody assays in some enrolling sites, or potential lack of familiarity by some treating clinicians. Despite this limitation, we were able to share reliable patient-level information for clinical characteristics and patient outcomes and to provide comparison with historical controls.

In conclusion, VTE events after SARS-CoV-2 vaccination are less frequently associated with traditional VTE risk factors than historical controls. VTE events after adenovirus-based vaccination, but not after mRNA-based vaccination, may occur more frequently as CVST or at multiple sites, and the latter are associated with higher mortality rates compared with historical controls.

## Figures and Tables

**Table 1:** Patient characteristics based on the type of COVID-19 vaccine.

	Adenovirus-Based Vaccines (Astrazeneca, Johnson and Johnsson)	mRNA-Based Vaccines (Pfizer and Moderna)	Controls from 2018–2019 ^a^
**Number of patients**	***28***	***74***	***911***
**Most common location of venous thrombosis**			
Pulmonary embolism with or without DVT	11 (39.3%)	48 (64.9%)	476 (52.3%)
Isolated DVT	7 (25.0%)	21 (28.4%)	405 (44.5%)
Cerebral venous sinus thrombosis only	3 (10.7%)	2 (2.7%)	4 (0.4%)
Splanchnic vein thrombosis only	2 (7.1%)	2 (2.7%)	14 (1.5%)
Venous thrombosis in >1 territory ^b^	5 (17.9%)	1 (1.4%)	12 (1.3%)
**Time of first vaccine dose (days)**	14.2 ± 1.1	22.8 ± 1.85	-
**Time of second vaccine dose (days)**	-	10.8 ± 2.7	-
**Sex**, women	11 (39.3%)	50 (67.6%)	470 (51.6%)
**Mean age—years (SEM)**	53.8 ± 2.53	78.3 ± 1.72	65.5 ± 0.56
**Age < 50 years N, %**	12 (42.9%)	6 (8.1%)	172 (18.9%)
**Major risk factors**			
Active cancer	1 (3.6%)	9 (12.2%)	136 (14.9%)
Major surgery within 30 days	1/27 (3.7%)	1 (1.4%)	105 (11.5%)
Medical hospitalization > 24hours within 30 days	0	1 (1.4%)	62 (6.8%)
Recent immobilization for ≥ 4 days within 30 days	2/27 (7.4%)	14 (18.9%)	192 (21.1%)
Pregnancy, puerperium, assisted reproductive therapy or contraceptive hormonal therapies	0	3 (4.1%)	74 (8.1%)
None of the above	24 (85.7%)	49 (66.2%)	462 (50.7%)
**Co-morbidities and other risk factors**			
Chronic lung disease	3 (10.7%)	5 (6.8%)	107 (11.7%)
Heart failure	1/26 (3.8%)	10/73 (13.7%)	57/905 (6.3%)
Coronary or peripheral arterial disease or ischemic stroke	3 (10.7%)	9 (12.2%)	116 (12.7%)
Hypertension	10 (35.7%)	37 (50.0%)	426 (46.8%)
Personal history of VTE	3/27 (11.1%)	15 (20.3%)	121 (13.3%)
Family history of VTE	3 (10.7%)	0	81 (8.9%)
Known thrombophilia ^c^	1/24 (4.2%)	2/62 (3.2%)	19/820 (2.3%)
Major bleeding in the past 30 days	1 (3.6%)	2 (2.7%)	23 (2.5%)
**Laboratory Tests**			
D-dimer (Positive) ^d^ (N = 746)	19/21 (100.0%)	55/56 (98.2%)	651/669 (97.3%)
D-dimer levels > 5× upper limit ^d^	9/21 (42.9%)	36/55 (65.5%)	402/651 (61.8%)
D-dimer levels > 10× upper limit ^d^	4/21 (19.0%)	28/55 (50.9%)	237/651 (36.4%)
Fibrinogen (mg/dL)	423 ± 41.0	393 ± 17.9	408 ± 5.35
Fibrinogen < 150 mg/dL	2 (7.1%)	0	3 (0.3%)
Abnormal INR (as reported by sites)	4 (14.3%)	6 (8.1%)	75 (8.2%)
INR values (N = 983)	1.02 ± 0.03	1.02 ± 0.01	(N = 884) 1.02 ± 0
Platelet count (/fL)	164.2 ± 19.07	226.3 ± 8.73	229.8 ± 2.95
Platelet count (/fL) Median (Q1, Q3)	192 (62–244)	218 (187–250)	217 (174–268)
Platelet count < 150,000/fL	11/27 (40.7%)	10 (13.5%)	134/910 (14.7%)
Platelet count < 50,000/fL	5/27 (18.5%)	0	1/910 (0.1%)
PF-4 antibody tested (Yes/No)	5/9 (55.6%)	0	0
PF-4 antibody above normal limit	5 (100.0%)	–	–
AST	(N = 16) 65.7 ± 23.0	(N = 43) 40.8 ± 9.5	(N = 459) 31.9 ± 1.9
ALT	(N = 15) 85.1 ± 38.4	(N = 51) 38.6 ± 9.68	(N = 596) 32.0 ± 1.54
SARS-CoV2 Status			
Infected	1 (3.6%)	4 (5.4%)	0
Not infected or not tested	27 (96.4%)	70 (94.6%)	911 (100.0%)
Countries			
Spain	17 (60.7%)	51 (68.9%)	701 (76.9%)
Austria	2 (7.1%)	1 (1.4%)	0
Czech Republic	2 (7.1%)	3 (4.1%)	2 (0.2%)
France	3 (10.7%)	3 (4.1%)	69 (7.6%)
Israel	0	6 (8.1%)	29 (3.2%)
Italy	4 (14.3%)	8 (10.8%)	56 (6.1%)
USA	0	2 (2.7%)	54 (5.9%)

^a^ Patients with thrombosis between 1 February 2018 to 22 April 2018 and 1 February 2019 to 22 April 2019 excluding superficial veins (DVT, PE, splanchnic vein thrombosis, or cerebral venous thrombosis) enrolled from centers that included post-vaccination cases ^b^ At least two of the following: (i) upper and/or lower extremity DVT and/or PE; (ii) CVST; and (iii) Splanchnic vein thrombosis. ^c^ Includes antiphospholipid antibody syndrome, inflammatory bowel disease, factor V Leiden, and prothrombin G 20210 mutation. ^d^ Laboratory tests were performed in each local institution. SEM: standard error of the mean. – means not applicable.

**Table 2:** Treatment Strategies.

	Adenovirus-Based Vaccines (Astrazeneca, Johnson and Johnsson)	mRNA-Based Vaccines (Pfizer and Moderna)	Controls from 2018–2019 ^a^
**Total Number of patients**	***28***	***74***	***911***
**Pulmonary Embolism or Deep Vein Thrombosis**	18	69	881
***Initial treatment***			
Unfractionated heparin	1 (5.6%)	1 (1.4%)	52 (5.9%)
Low-molecular weight heparin	8 (44.4%)	62 (89.9%)	713 (80.9%)
Argatroban/bivalirudin/danaparoid/fondaparinux	2 (11.1%)	0	15 (1.7%)
Direct oral anticoagulants	3 (16.7%)	1 (1.4%)	78 (8.9%)
No anticoagulation	4 (22.2%)	4 (5.8%)	6 (0.7%)
Vitamin K antagonists	0	0	2 (0.2%)
Fibrinolytic therapy	0	1 (1.4%)	15 (1.7%)
Surgical or percutaneous thrombectomy	0	1 (1.4%)	10 (1.1%)
Inferior vena cava filters	0	1 (1.4%)	23 (2.6%)
**CVST**	*3*	*2*	*4*
***Initial treatment***			
Unfractionated heparin	0	0	1 (25.0%)
Low-molecular weight heparin	2 (66.7%)	2 (100.0%)	2 (50.0%)
Argatroban/bivalirudin/danaparoid/fondaparinux	1 (33.3%)	0	0
Direct oral anticoagulants	0	0	0
No anticoagulation	0	0	1 (25.0%)
Fibrinolytic therapy	0	0	0
**Splanchnic Vein Thrombosis**	*2*	*2*	*14*
***Inpatient treatment***			
Unfractionated heparin	0	0	1 (7.1%)
Low-molecular weight heparin	1 (50%)	2 (100%)	12 (85.7%)
Argatroban/bivalirudin/danaparoid/fondaparinux	0	0	0
Direct oral anticoagulants	0	0	0
No anticoagulation	1 (50%)	0	1 (7.4%)
Fibrinolytic therapy	0	0	0
**Thrombosis At Multiple Sites ^b^**	*5*	*1*	*12*
***Inpatient treatment***			
Unfractionated heparin	0	0	2 (16.7%)
Low-molecular weight heparin	3 (60.0%)	1 (100.0%)	6 (50.0%)
Argatroban/bivalirudin/danaparoid/fondaparinux	1 (20.0%)	0	0
Direct oral anticoagulants	1 (20.0%)	0	2 (16.7%)
No anticoagulation	0	0	1 (8.3%)
Vitamin K antagonists	0	0	1 (8.3%)
Fibrinolytic therapy	0	0	0

^a^ Patients with thrombosis between 1 February 2018 to 22 April 2018 and 1 February 2019 to 22 April 2019 excluding superficial veins (DVT, PE, splanchnic vein thrombosis, or cerebral venous thrombosis) enrolled from centers that included post-vaccination. ^b^ At least two of the following: (i) upper and/or lower extremity DVT and/or PE, (ii) CVST, and (iii) Splanchnic vein thrombosis.

**Table 3:** Clinical Outcomes.

	Adenovirus-Based Vaccines (Astrazeneca, Johnson and Johnsson)	mRNA-Based Vaccines (Pfizer and Moderna)	Controls from 2018–2019 ^a^
Total Number of patients	***28***	***74***	***911***
All-cause mortality N, %	4 (14.3%)	1 (1.4%)	6 (0.7%)
Unadjusted odds ratio	25.14 (6.66–94.91)	2.07 (0.25–17.39)	Ref.
Adjusted odds ratio ^b^	13.24 (1.44–121.3)	1.05 (0.11–10.16)	Ref.
Major bleeding N, %	3 (10.7%)	0	9 (1.0%)
Unadjusted odds ratio	12.03 (3.07–47.13)	-	Ref.
Adjusted odds ratio ^b^	9.03 (1.07–76.13)	-	Ref.
Pulmonary Embolism or Deep Vein Thrombosis	***18***	***69***	***881***
All-cause mortality N, %	0	1 (1.4%)	5 (0.6%)
Unadjusted odds ratio	-	2.58 (0.30–22.37)	Ref.
Adjusted odds ratio	-	1.56 (0.18–13.77)	Ref.
Major bleeding N, %	0	0	9 (1.0%)
Unadjusted odds ratio	-	-	Ref.
Adjusted odds ratio	-	-	Ref.
CVST	***3***	***2***	***4***
All-cause mortality N, %	2 (66.7%)	0	0
Major bleeding N, %	1 (33.3%)	0	0
Unadjusted odds ratio	-	-	-
Splanchnic Vein Thrombosis	***2***	***2***	***14***
All-cause mortality N, %	0	0	0
Major bleeding N, %	0	0	0
Unadjusted odds ratio	-	-	-
Thrombosis At Multiple Sites ^c^	***5***	***1***	***12***
All-cause mortality N, %	2 (40.0%)	0	1 (8.3%)
Unadjusted odds ratio	7.33 (0.48–111.2)	-	Ref.
Major bleeding N, %	2 (40.0%)	0	0

^a^ Patients with thrombosis between 1 February 2018 to 30 April 2018 and 1 February 2019 to 30 April 2019 excluding superficial veins (DVT, PE, splanchnic vein thrombosis, or cerebral venous thrombosis) enrolled from centers that included post-vaccination. These patients were selected as the reference group. ^b^ For adjustment, mixed effects models were used with age, sex, thrombocytopenia, cerebral or splanchnic vein involvement, cancer, and history of VTE as covariates, and enrolling center as a random effect. ^c^ At least two of the following: (i) upper and/or lower extremity DVT and/or PE, (ii) CVST, and(iii) Splanchnic vein thrombosis. - means not applicable.

## Data Availability

Data available on request due to privacy restrictions.

## Supplementary Materials

The following are available online at https://www.mdpi.com/article/10.3390/v14020178/s1, Table S1: Clinical and laboratory characteristics of patients with venous thrombosis (VTE) within 30 days of receiving a COVID-19 vaccine; Table S2: Patient characteristics based on specific vaccine; Table S3: Clinical characteristics of patients with VTE and thrombocytopenia (Platelet Count < 150,000/fL) after vaccination against SARS-CoV2.

## Appendix A

Coordinator of the RIETE Registry: Manuel Monreal.

RIETE Steering Committee Members: Paolo Prandoni, Benjamin Brenner and Dominique Farge-Bancel.

RIETE National Coordinators: Raquel Barba (Spain), Pierpaolo Di Micco (Italy), Laurent Bertoletti (France), Sebastian Schellong (Germany), Inna Tzoran (Israel), Abilio Reis (Portugal), Marijan Bosevski (R. North Macedonia), Henri Bounameaux (Switzerland), Radovan Malý (Czech Republic), Peter Verhamme (Belgium), Joseph A. Caprini (USA), Hanh My Bui (Vietnam).

RIETE Registry Coordinating Center: S & H Medical Science Service.

Members of the RIETE Group

**SPAIN:** Adarraga MD, Aibar J, Amado C, Arcelus JI, Asuero A, Ballaz A, Barba R, Barbagelata C, Barrón M, Barrón-Andrés B, Blanco-Molina A, Beddar Chaib F, Botella E, Castro J, Chasco L, Criado J, de Ancos C, de Cossío S, del Toro J, Demelo-Rodríguez P, Díaz-Brasero AM, Díaz-Pedroche MC, Díaz-Peromingo JA, Di Campli MV, Dubois-Silva A, Escribano JC, Espósito F, Farfán-Sedano AI, Fernández-Capitán C, Fernández-Reyes JL, Fidalgo MA, Flores K, Font C, Font L, Francisco I, Gabara C, Galeano-Valle F, García MA, García-Bragado F, García de Herreros M, García de la Garza R, García-Díaz C, Gil-Díaz A, Gómez-Cuervo C, González-Aragonés E, Grau E, Guirado L, Gutiérrez J, Hernández-Blasco L, Jara-Palomares L, Jaras MJ, Jiménez D, Jiménez R, Jiménez-Alfaro C, Joya MD, Lainez-Justo S, Lalueza A, Latorre A, Lecumberri R, León-Ramírez JM, Lima J, Lobo JL, López-Jiménez L, López-Miguel P, López-Núñez JJ, López-Reyes R, López-Sáez JB, Lorenzo A, Madridano O, Maestre A, Marchena PJ, Martín del Pozo M, Martín-Martos F, Martínez-Urbistondo D, Mella C, Mercado MI, Moisés J, Monreal M, Muñoz-Blanco A, Nieto JA, Núñez-Fernández MJ, Olid-Velilla M, Olivares MC, Osorio J, Otalora S, Otero R, Paredes D, Pedrajas JM, Pérez-Jacoiste MA, Porras JA, Portillo J, Rodríguez-Galán I, Rodríguez-Matute C, Rosa V, Ruiz-Artacho P, Ruiz-Ruiz J, Salgueiro G, Sánchez-Martínez R, Sánchez-Muñoz-Torrero JF, Sancho T, Soler S, Suárez-Rodríguez B, Suriñach JM, Torres MI, Tolosa C, Trujillo-Santos J, Uresandi F, Valero B, Valle R, Varona JF, Vela L, Vela JR, Vidal G, Villalobos A, Villares P, Zamora C, **AUSTRIA:** Ay C, Nopp S, Pabinger I, **BELGIUM:** Martens C, Vanassche T, Verhamme P, **CZECH REPUBLIC:** Hirmerova J, Malý R, Varhaník F, FRANCE: Accassat S, Ait Abdallah N, Bertoletti L, Bura-Riviere A, Catella J, Couturaud F, Crichi B, Debourdeau P, Espitia O, Farge-Bancel D, Grange C, Helfer H, Lacut K, Le Mao R, Mahé I, Morange P, Moustafa F, Poenou G, Sarlon-Bartoli G, Suchon P, Quere I, **GERMANY:** Schellong S, **ISRAEL:** Braester A, Brenner B, Kenet G, Tzoran I, **ITALY:** Basaglia M, Bilora F, Bortoluzzi C, Brandolin B, Ciammaichella M, De Angelis A, Di Micco P, Imbalzano E, Merla S, Pesavento R, Prandoni P, Siniscalchi C, Tufano A, Visonà A, Vo Hong N, Zalunardo B, **JAPAN:** Nishimoto Y, Sato Y, **LATVIA:** Birzulis J, Kigitovica D, Skride A, **PORTUGAL:** Fonseca S, Martins F, Meireles J, **REPUBLIC OF NORTH MACEDONIA:** Bosevski M, **SWITZERLAND:** Bounameaux H, Mazzolai L, **USA:** Bikdeli B, Caprini JA, **VIETNAM:** Bui HM.
