# Supplementary material for: Venous Thrombosis within 30 Days after Vaccination against SARS-CoV-2 in a Multinational Venous Thromboembolism Registry
Source: Viruses. 2022 Jan 18;14(2):178. doi: 10.3390/v14020178 (PMC8878689; doi:10.3390/v14020178)
Supplement: Supplementary file 1 [file viruses-14-00178-s001.zip › viruses-1501247-supplementary.pdf]

Article

Venous Thrombosis within 30 Days After Vaccination Against SARS-CoV-2 in a Multinational Venous Thromboembolism Registry

supplementary materials

MDPI

Table S1. Clinical and Laboratory Characteristics of Patients with Venous Thrombosis (VTE) Within 30 days of Receiving a COVID-19 Vaccine

Patient	Age (years)	Sex (F/M)	Type of Thrombotic Event [a]	Vaccine Name	Time since First Vaccine Dose (days)	Time since Second Vaccine Dose (days)	History of Venous Thrombosis (Yes/ No)	D-dimer	Fibrinogen mg/dL	INR	Platelet count x1000/fL)	PF-4 Antibody	AST	ALT	SARS-CoV2 Status (infected, not infected, unknown)	Antithrombotic regimen prior to VTE [a]	Management	14-day Outcome
1	37	M	Cerebral vein sinus thrombosis	Pfizer	42	18	Yes	D-dimer levels >3 x upper limit	321	0.99	192	Not done	28	35	Not infected		Low-molecular weight heparin	
2	50	F	Pulmonary embolism with DVT in vena cava	Astrazeneca	19		No	Not done	691	0.98	62	Not done	14	16	Not infected		Low-molecular weight heparin	
3	47	F	Pulmonary embolism	Pfizer	39	13	No	D-dimer levels >4 x upper limit	241	1.08	236	Not done	64	140	Not infected		Fibrinolytic therapy	
4	31	M	DVT in portal and cerebral veins	Astrazeneca	17		No	Not done	160	1.28	33	Positive	159	283	Not infected		Low-molecular weight heparin	
5	92	F	Pulmonary embolism with Lower limb DVT	Pfizer	8		No	D-dimer levels >32 x upper limit	600	1.00	218	Not done	28	20	Not infected	Aspirin 100 mg/d	Low-molecular weight heparin	
6	98	F	Lower limb DVT	Pfizer	5	-16	No	Not done	679	1.00	248	Not done		13	Not infected		Low-molecular weight heparin	
7	56	F	PE and DVT in lower extremities, portal vein, cerebral vein, vena cava and jugular vein	Astrazeneca	7		No	D-dimer levels less than upper limit	440	1.01	32	Positive			Not infected		Fondaparinux, Immunoglobulins	Major bleeding and death

8	80	F	Pulmonary embolism	Pfizer	14	-7	Yes	D-dimer levels >8 x upper limit	532	1.03	207	Not done			Not infected		Low-molecular weight heparin	
9	90	F	Lower limb DVT	Pfizer	12		No	D-dimer levels >4 x upper limit	516	0.96	346	Not done	67	20	Not infected		Low-molecular weight heparin	
10	44	M	Pulmonary embolism	Astraze neca	17		No	D-dimer levels >9 x upper limit	235	1.02	186	Not done	30	17	Not infected		Low-molecular weight heparin	
11	83	F	Pulmonary embolism	Pfizer	47	15	No	D-dimer levels >37 x upper limit	583	0.94	218	Not done	19	12	Not infected		Low-molecular weight heparin	
12	98	F	Lower limb DVT	Pfizer	30	9	No	D-dimer levels >20 x upper limit	332	0.92	227	Not done			Not infected		Low-molecular weight heparin	Death
13	81	M	Lower limb DVT	Pfizer	19		No	D-dimer levels >10 x upper limit	564	0.97	233	Not done		11	Not infected		Low-molecular weight heparin	
14	88	M	Pulmonary embolism	Pfizer	26	4	No	D-dimer levels >10 x upper limit	608	1.00	190	Not done		9	Not infected		Low-molecular weight heparin	
15	44	F	Upper extremity DVT	Pfizer	23		No	D-dimer levels >1 x upper limit	217	1.05	220	Not done	105	121	Not infected		Low-molecular weight heparin	
16	84	F	Pulmonary embolism with Lower limb DVT	Modern a	12		Yes	D-dimer levels >10 x upper limit	700	1.07	205	Not done	20	23	Not infected		Low-molecular weight heparin	
17	45	M	Cerebral vein sinus thrombosis	Astraze neca	24		No	D-dimer levels less than upper limit	600	0.98	125	Positive			Not infected		Low-molecular weight heparin, Immunoglobuli ns	Major bleeding and death

18	42	M	Lower limb DVT	Astrazeneca	8		Yes	D-dimer levels >9 x upper limit	720	0.91	309	Not done	15	22	Not infected		Low-molecular weight heparin	
19	89	F	Pulmonary embolism	Pfizer	29		No	D-dimer levels >4 x upper limit	576	1.04	299	Not done	48	78	Not infected	Anticoagulants, Vitamin K antagonists	Low-molecular weight heparin	
20	81	F	DVT in ovarian vein	Moderna	28		Yes	D-dimer levels >10 x upper limit	310	1.01	335	Not done	33	35	Not infected		Biosimilars of enoxaparin	
21	67	F	Pulmonary embolism	Pfizer	20		No	Not done	913	1.30	250	Not done	25	15	Not infected	Antiplatelet, Aspirin 100 mg/d	Low-molecular weight heparin	
22	90	F	Lower limb DVT	Pfizer	6	-15	No	Not done	257	0.98	135	Not done			Not infected		Low-molecular weight heparin	
23	66	F	Pulmonary embolism	Pfizer	29	8	No	Not done	639	0.90	355	Not done	22	29	Not infected	Antiplatelet, Aspirin 100 mg/d	Low-molecular weight heparin	
24	64	M	Pulmonary embolism	Astrazeneca	10		No	D-dimer levels >1 x upper limit	993	0.93	194	Not done			Not infected			
25	92	F	Pulmonary embolism	Pfizer	18		No	D-dimer levels >9 x upper limit	504	0.96	190	Not done		20	Not infected		Low-molecular weight heparin	
26	85	F	Pulmonary embolism	Pfizer	23		No	D-dimer levels >27 x upper limit	247	1.04	165	Not done	417	477	Not infected		Low-molecular weight heparin	
27	84	F	Pulmonary embolism	Pfizer	14		Yes	D-dimer levels >2 x upper limit	296	NA	233	Not done			Not infected	Antiplatelet, Aspirin 100 mg/d		
28	73	M	Lower limb DVT	Pfizer	2		No	Unknown	211	0.92	75	Not done	25	14	Not infected		Biosimilars of enoxaparin	

29	87	F	Pulmonary embolism with Lower limb DVT	Pfizer	14		No	D-dimer levels >8 x upper limit	367	1.08	201	Not done			Not infected		Low-molecular weight heparin	
30	63	F	Pulmonary embolism	Astrazeneca	11		No	D-dimer levels >2 x upper limit	675	0.95	149	Positive	22	11	Not infected		Fondaparinux	
31	51	F	Cerebral vein sinus thrombosis	Pfizer	45	24	No	Positive, Unknown levels	516	0.91	329	Not done			Not infected		Low-molecular weight heparin	
32	84	M	Pulmonary embolism	Pfizer	29	0	No	Unknown	310	1.04	229	Not done			Not infected		Low-molecular weight heparin	
33	75	F	Pulmonary embolism	Pfizer	4		No	D-dimer levels >14 x upper limit	426	0.94	414	Not done			Not infected			
34	87	M	Lower limb DVT	Pfizer	54	33	No	Unknown	286	1.09	188	Not done	40	67	Not infected	Antiplatelet, Aspirin 300 mg/d	Low-molecular weight heparin	
35	85	F	Lower limb DVT	Pfizer	34	8	No	D-dimer levels >38 x upper limit	411	1.07	114	Not done	48	84	Not infected		Low-molecular weight heparin	
36	87	F	Pulmonary embolism	Pfizer	18		No	D-dimer levels >12 x upper limit	282	1.00	318	Not done			Not infected		Low-molecular weight heparin	
37	41	M	Pulmonary embolism with DVT	Astrazeneca	21		No	D-dimer levels >7 x upper limit	480	0.96	192	Not done	18	30	Not infected		Low-molecular weight heparin	
38	70	F	Pulmonary embolism	Pfizer	56	25	No	D-dimer levels >52 x upper limit	307	1.00	95	Not done		7	Not infected		Low-molecular weight heparin	
39	92	M	Pulmonary embolism	Pfizer	36	12	No	D-dimer levels >3 x upper limit	445	0.91	140	Not done			Not infected		Low-molecular weight heparin	

40	90	M	Pulmonary embolism	Pfizer	28	7	No	D-dimer levels >46 x upper limit	379	0.98	153	Not done		28	Not infected		Low-molecular weight heparin	
41	91	M	Pulmonary embolism	Moderna	35	17	No	D-dimer levels >12 x upper limit	301	1.00	150	Not done			Not infected		Low-molecular weight heparin	
42	84	F	Pulmonary embolism	Pfizer	27		No	D-dimer levels >4 x upper limit	417	0.94	191	Not done	29	19	Not infected		Low-molecular weight heparin	
43	73	F	Pulmonary embolism	Pfizer	54	33	No	Not done	537	1.02	461	Not done	26	21	Not infected	Antiplatelet, Aspirin 100 mg/d	Low-molecular weight heparin	
44	83	M	Pulmonary embolism with Lower limb DVT	Pfizer	5		No	D-dimer levels >18 x upper limit	396	1.09	395	Not done	18	6	Not infected	Antiplatelet, Aspirin 300 mg/d	Low-molecular weight heparin	
45	45	F	Cerebral vein sinus thrombosis	Astrazeneca	15		Unspecified	Positive, Unknown levels	393	1.07	47	Not done	99	121	Not infected		Low-molecular weight heparin	
46	90	F	Pulmonary embolism	Pfizer	8		Yes	Positive, Unknown levels	460	1.04	140	Not done			Not infected		Low-molecular weight heparin	
47	88	F	Lower and upper extremity DVT	Pfizer	46	27	Yes	Not done	310	1.20	242	Not done	66	35	Not infected	Antiplatelet, Aspirin 100 mg/d	Low-molecular weight heparin	
48	75	M	Pulmonary embolism	Pfizer	7		No	D-dimer levels >7 x upper limit	311	0.92	231	Not done			Infected		Low-molecular weight heparin	
49	33	F	Pulmonary embolism	Pfizer	45	23	No	Not done	329	0.97	258	Not done	10	12	Infected		Low-molecular weight heparin	
50	78	F	Pulmonary embolism with DVT	Pfizer	7		No	D-dimer levels >2 x upper limit	253	1.07	220	Not done	44	43	Infected		Biosimilars of enoxaparin	

51	82	M	Pulmonary embolism with Lower limb DVT	Pfizer	35	14	No	D-dimer levels >7 x upper limit	261	1.00	249	Not done	17	10	Not infected		Low-molecular weight heparin	
52	82	F	Pulmonary embolism	Pfizer	18		Yes	D-dimer levels >41 x upper limit	237	1.01	247	Not done	22	21	Not infected	Antiplatelet, Aspirin 100 mg/d	Low-molecular weight heparin	
53	76	F	Pulmonary embolism with Lower limb DVT	Moderna	5		Yes	D-dimer levels >20 x upper limit	292	1.07	155	Not done	20	16	Not infected		Low-molecular weight heparin	
54	75	F	Lower limb DVT	Pfizer	6		No	D-dimer levels >28 x upper limit	347	0.99	130	Not done			Not infected			
55	78	M	Pulmonary embolism with Lower limb DVT	Pfizer	8		No	D-dimer levels >32 x upper limit	358	0.92	160	Not done		17	Not infected		Low-molecular weight heparin	
56	81	M	Pulmonary embolism	Moderna	16		No	D-dimer levels >4 x upper limit	442	1.05	247	Not done			Not infected	Antiplatelet, Aspirin 100 mg/d	Low-molecular weight heparin	
57	77	M	Lower limb DVT	Pfizer	12		Yes	D-dimer levels >20 x upper limit	398	1.23	187	Not done	18	11	Not infected		Biosimilars of enoxaparin	
58	49	F	PE and DVT in portal, mesenteric, splenic and cerebral veins	Astrazeneca	10		No	Positive, Unknown levels	101	1.01	18	Not done	46	33	Not infected		Low-molecular weight heparin	Major bleeding and death
59	38	F	Lower limb DVT	Pfizer	2	-33	Yes	D-dimer levels >2 x upper limit	234	0.96	217	Not done	18	18	Not infected		Low-molecular weight heparin	
60	40	F	Pulmonary embolism	Moderna	8		No	Unknown	266	1.08	329	Not done	17	12	Not infected		Direct oral anticoagulants	
61	53	M	Lower limb DVT	Astrazeneca	12		No	Positive, Unknown levels	444	1.04	325	Not done	34	41	Not infected		Direct oral anticoagulants	

62	63	M	DVT in portal vein	Astrazeneca	13		No	D-dimer levels >7 x upper limit	631	NA	121	Positive	368	560	Infected			
63	62	M	Pulmonary embolism with Lower limb DVT	Pfizer	8		No	Not done	202	1.30	246	Not done	36	49	Not infected		Unfractionated heparin	
64	35	M	Cerebral vein sinus thrombosis	Astrazeneca	14		No	D-dimer levels >59 x upper limit	101	?	34	Not done			Not infected		Fondaparinux	Death
65	57	M	Pulmonary embolism with DVT in lower extremities	Moderna	11		Yes	D-dimer levels >14 x upper limit	237	0.92	215	Not done	17	18	Not infected	Anticoagulants, Vitamin K antagonists	Low-molecular weight heparin	
66	88	F	Pulmonary embolism	Pfizer	57	29	No	D-dimer levels >29 x upper limit	441	1.00	191	Not done	23	17	Not infected		Low-molecular weight heparin	
67	65	F	Pulmonary embolism	Astrazeneca	20		Yes	D-dimer levels >3 x upper limit	248	0.91	284	Not done	17	12	Not infected	Antiplatelet, Aspirin 100 mg/d	Unfractionated heparin	
68	87	F	Lower limb DVT	Pfizer	20		No	Not done	445	1.02	258	Not done	28	22	Not infected		Biosimilars of enoxaparin	
69	82	F	Lower limb DVT	Moderna	5	-23	No	D-dimer levels >7 x upper limit	620	1.04	233	Not done			Not infected	Antiplatelet, Aspirin 100 mg/d	Low-molecular weight heparin	
70	74	F	Pulmonary embolism with DVT	Pfizer	7		No	D-dimer levels >20 x upper limit	234	0.91	135	Not done	90	116	Not infected		Biosimilars of enoxaparin	
71	83	M	Lower limb DVT	Pfizer	13		Yes	D-dimer levels >12 x upper limit	435	0.99	132	Not done	18	13	Not infected		Low-molecular weight heparin	

72	83	M	Pulmonary embolism	Moderna	4		No	D-dimer levels >2 x upper limit	648	1.02	193	Not done	75	36	Not infected		Biosimilars of enoxaparin	
73	47	M	PE and DVT in lower extremities and cerebral vein	Astrazeneca	10		No	Positive, Unknown levels	323	0.92	51	Not done			Not infected		Low-molecular weight heparin	
74	85	F	Lower limb DVT	Pfizer	16	-5	Yes	D-dimer levels >9 x upper limit	517	1.09	185	Not done			Not infected		Low-molecular weight heparin	
75	55	M	Lower limb DVT	Pfizer	29	8	No	Negative	396	0.96	202	Not done			Not infected		Low-molecular weight heparin	
76	90	M	Pulmonary embolism	Moderna	51	23	No	Not done	275	1.10	251	Not done	11	17	Infected		Low-molecular weight heparin	
77	62	M	Lower limb DVT	Astrazeneca	12		No	D-dimer levels >8 x upper limit	564	0.99	216	Not done			Not infected	Antiplatelet, Aspirin 300 mg/d	Low-molecular weight heparin	
78	82	M	Pulmonary embolism	Astrazeneca	8		No	D-dimer levels >4 x upper limit	383	0.97	223	Not done	32	35	Not infected	Antiplatelet, Aspirin 100 mg/d		
79	92	F	Lower limb DVT	Pfizer	32	11	No	D-dimer levels >14 x upper limit	284	1.07	285	Not done			Not infected		Biosimilars of enoxaparin	
80	72	F	Lower limb DVT	Moderna	6		No	D-dimer levels >13 x upper limit	249	1.00	381	Not done	38	40	Not infected	Antiplatelet, Aspirin 100 mg/d	Biosimilars of enoxaparin	
81	76	M	Pulmonary embolism	Pfizer	12		No	D-dimer levels >35 x upper limit	221	0.94	175	Not done	21	21	Not infected		Biosimilars of enoxaparin	
82	63	F	Pulmonary embolism with Lower limb DVT	Astrazeneca	18		No	D-dimer levels >15 x upper limit	202	1.06	65	Not done			Not infected		Low-molecular weight heparin	

83	93	F	Pulmonary embolism	Pfizer	32	11	No	Positive, Unknown levels	340	1.06	187	Not done	25	19	Not infected		Low-molecular weight heparin	
84	88	F	Pulmonary embolism	Pfizer	48	27	No	Unknown	215	0.98	267	Not done	13	24	Not infected	Antiplatelet, Aspirin 100 mg/d	Low-molecular weight heparin	
85	89	F	Pulmonary embolism with Lower limb DVT	Pfizer	21		No	D-dimer levels >20 x upper limit	304	1.00	210	Not done			Not infected			
86	64	M	Pulmonary embolism with Lower limb DVT	Astrazeneca	13		No	D-dimer levels >20 x upper limit	268	0.98	209	Not done			Not infected			
87	77	F	Pulmonary embolism	Pfizer	18		No	D-dimer levels >7 x upper limit	343	0.92	271	Not done			Not infected		Low-molecular weight heparin	
88	67	F	Pulmonary embolism	Astrazeneca	4		No	Unknown	343	1.04		Not done			Not infected			
89	73	F	Pulmonary embolism	Astrazeneca	13		No	Positive, Unknown levels	381	0.90	214	Not done	14	16	Not infected		Direct oral anticoagulants	
90	26	F	Lower limb DVT	Astrazeneca	30		Yes	Unknown	384	1.02	249	Not done			Not infected		Fondaparinux	
91	80	F	Pulmonary embolism	Pfizer	45	17	Yes	Positive, Unknown levels	383	0.97	204	Not done			Not infected		Low-molecular weight heparin	
92	67	M	Lower limb DVT	Astrazeneca	21		No	Unknown	323	1.03	216	Not done	24	48	Not infected		Direct oral anticoagulants	
93	86	F	Pulmonary embolism	Pfizer	38	10	No	Unknown	435	0.94	378	Not done	18	14	Not infected		Low-molecular weight heparin	
94	77	F	Pulmonary embolism	Pfizer	8		No	D-dimer levels >2 x upper limit	524	0.93	230	Not done	18	11	Not infected	Antiplatelet, Aspirin 100 mg/d	Low-molecular weight heparin	
95	64	M	DVT in portal vein	Astrazeneca	9		No	Unknown	319	1.60	244	Not done	132		Not infected		Low-molecular weight heparin	

96	73	F	DVT in portal and cerebral veins	Pfizer	6		No	Positive, Unknown levels	231	1.10	204	Not done			Not infected	Antiplatelet, Aspirin 100 mg/d	Low-molecular weight heparin	
97	60	M	Lower limb DVT	Astrazeneca	8		No	Unknown	803	1.00	250	Not done			Not infected		Low-molecular weight heparin	
98	90	F	Pulmonary embolism	Pfizer	34	12	No	Positive, Unknown levels	264	0.97	148	Not done	15	29	Not infected	Antiplatelet, Aspirin 100 mg/d	Low-molecular weight heparin	
99	85	M	DVT in portal vein	Pfizer	50	28	No	Not done	833	1.38	185	Not done		9	Not infected		Low-molecular weight heparin	
100	92	M	Lower limb DVT	Pfizer	8		No	Unknown	315	0.96	212	Not done	16	14	Not infected	Antiplatelet, Aspirin 300 mg/d	Biosimilars of enoxaparin	
101	42	F	Lower limb DVT	Astrazeneca	18		No	D-dimer levels >4 x upper limit	309	1.06	320	Not done			Not infected		Low-molecular weight heparin	
102	43	M	DVT in lower extremities and cerebral vein	Astrazeneca	15		No	D-dimer levels >40 x upper limit	327	0.96	64	Not done	28	31	Not infected		Direct oral anticoagulants	

a: includes aspirin, other antiplatelet agents, anticoagulants b: Some patients had more than 1 type of thrombotic event diagnosed.

Table S2. Patient Characteristics Based on Specific Vaccine				
	**ChAdOx1 nCov-19 (AstraZeneca)**	**mRNA-1273 (Moderna)**	**BNT162b2 (Pfizer)**	**Controls from 2018-2019** [a]
**Number of patients**	**28**	**11**	**63**	**911**
**Most common location of venous thrombosis**				
Pulmonary embolism with or without DVT	11 (39.3%)	8 (72.7%)	40 (63.5%)	476 (52.3%)
Isolated DVT	7 (25.0%)	2 (18.2%)	19 (30.2%)	405 (44.5%)
Cerebral venous sinus thrombosis only	3 (10.7%)	0	2 (3.2%)	4 (0.4%)
Splanchnic vein thrombosis only	2 (7.1%)	1 (9.1%)	1 (1.6%)	14 (1.5%)
Venous thrombosis in >1 territory [b]	5 (17.9%)	0	1 (1.6%)	12 (1.3%)
**Time of first vaccine dose (days)**	14.2±1.1	16.5±4.59	23.9±2	0±0
**Time of last vaccine dose (days)**	0±0	5.67±14.4	11.3±2.71	0±0
**Women**	11 (39.3%)	6 (54.5%)	44 (69.8%)	470 (51.6%)
**Mean age –years (SEM)**	53.8±2.53	76.1±4.57	78.7±1.86	65.5±0.56
**Age < 50 years N, %**	12 (42.9%)	1 (9.1%)	5 (7.9%)	172 (18.9%)
**Major risk factors**				
Active cancer	1 (3.6%)	2 (18.2%)	7 (11.1%)	136 (14.9%)
Major surgery within 30 days	1/27 (3.7%)	0	1/63 (1.6%)	105 (11.5%)
Medical hospitalization>24hours within 30 days	0	0	1 (1.6%)	62 (6.8%)
Recent immobilization for ≥4 days within 30 days *(N=1,012)*	2/27 (7.4%)	1 (9.1%)	13 (20.6%)	192 (21.1%)
Pregnancy, puerperium, assisted reproductive therapy or contraceptive hormonal therapies	0	0	3 (4.8%)	74 (8.1%)
None of the above	24 (85.7%)	8 (72.7%)	41 (65.1%)	462 (50.7%)
**Co-morbidities and other risk factors**				
Chronic lung disease	3 (10.7%)	1 (9.1%)	4 (6.3%)	107 (11.7%)
Heart failure	1/26 (3.8%)	1 (9.1%)	9/62 (14.5%)	57/905 (6.3%)
Coronary or peripheral arterial disease or ischemic stroke	3 (10.7%)	2 (18.2%)	7 (11.1%)	116 (12.7%)
Hypertension	10 (35.7%)	7 (63.6%)	30 (47.6%)	426 (46.8%)
Personal history of VTE	3/27 (11.1%)	4 (36.4%)	11 (17.5%)	121 (13.3%)
Family history of VTE	3 (10.7%)	0	0	81 (8.9%)
Known thrombophilia [c]	1/24 (4.2%)	1/9 (11.1%)	1/53 (1.9%)	19/820 (2.3%)
Major bleeding in the past 30 days	1 (3.6%)	0	2 (3.2%)	23 (2.5%)
**Laboratory Tests**				
D-dimer (Positive)[d]	21/21 (100.0%)	9/9 (100.0%)	46/47 (97.9%)	651/669 (97.3%)
D-dimer levels >5 x upper limit [d]	9/21 (42.9%)	7/9 (77.8%)	29/46 (63.0%)	402/651 (61.8%)
D-dimer levels >10 x upper limit [d]	4/21 (19.0%)	6/9 (66.7%)	22/46 (47.8%)	237/651 (36.4%)
Fibrinogen	423±41.0	395±53.4	393±19.0	408±5.35
Fibrinogen < 150	2 (7.1%)	0	0	3 (0.3%)
INR *(N=983)*	1.02±0.03	1.03±0.01	1.02±0.01	1.02±0
Platelet count (/fL)	164±19.1	245±22.6	223±9.47	230±2.95
Platelet count (/fL) Median (Q1, Q3)	192 (62-244)	233 (193-329)	217 (185-249)	217 (174-268)
Platelet count < 150,000/ fL	11/27 (40.7%)	0	10 (15.9%)	134/910 (14.7%)
Platelet count < 50/ μL	5/27 (18.5%)	0	0	1 (0.1%)
PF-4 antibody tested (Yes/No)	5 (55.6%)	0	0	0
PF-4 antibody above normal limit	5 (100.0%)	0	0	0
AST *(N=518)*	65.7±23.0	28.9±7.30	43.6±11.6	31.9±1.90
ALT *(N=662)*	85.1±38.4	24.6±3.81	41.1±11.4	32.0±1.54
**SARS-CoV2 Status**				
Infected	1 (3.6%)	1 (9.1%)	3 (4.8%)	0
Not infected	27 (96.4%)	10 (90.9%)	60 (95.2%)	911 (100.0%)
Not tested	0	0	0	0

a: Patients with thrombosis between 02/01/2018 to 4/22/2018 and 02/01/2019 to 4/22/2019 excluding superficial veins (DVT, PE, splanchnic vein thrombosis, or cerebral venous thrombosis) enrolled from centers that included post-vaccination cases b:

At least two of the following: i) upper and/or lower extremity DVT and/or PE; ii) CVST; iii) Splanchnic vein thrombosis. c: Includes antiphospholipid antibody syndrome, inflammatory bowel disease, factor V Leiden, prothrombin G 20210 mutation d: Laboratory tests were performed in each local institution. SEM: standard error of the mean.

**Table S3. Clinical Characteristics of Patients with VTE And Thrombocytopenia (Platelet Count < 150,000/ fL) after Vaccination Against SARS-CoV2**	
**Number of patients**	**21**
**Type of Vaccine (N)**	
ChAdOx1 nCov-19 (AstraZeneca)	11 (52.4%)
BNT162b2 (Pfizer)	10 (47.6%)
mRNA-1273 (Moderna)	0
Ad26.COV2.S (Johnson & Johnson/Janssen)	0
**Location of venous thrombosis**	
Pulmonary embolism with or without DVT	7 (33.3%)
Isolated DVT	5 (23.8%)
Cerebral venous sinus thrombosis only	3 (14.3%)
Splanchnic vein thrombosis only	1 (4.8%)
Venous thrombosis in >1 territory [a]	5 (23.8%)
**Time of first vaccine dose (days)**	17±2.86
**Time of last vaccine dose (days)**	8.4±6.52
**Women**	12 (57.1%)
**Mean age –years (SEM)**	64.24±4.31
**Age < 50 years N, %**	7 (33.3%)
**Major risk factors**	
Active cancer	2 (9.5%)
Major surgery within 30 days *(N=20)*	0
Medical hospitalization>24hours within 30 days	0
Recent immobilization for ≥4 days within 30 days *(N=20)*	4/20 (20.0%)
Pregnancy, puerperium, assisted reproductive therapy or contraceptive hormonal therapies	2 (9.5%)
None of the above	15 (71.4%)
**Co-morbidities and other risk factors**	
Chronic lung disease	3 (14.3%)
Heart failure *(N=20)*	1/20 (5.0%)
Coronary or peripheral arterial disease or ischemic stroke	0
Diabetes	0
Hypertension	8 (38.1%)
Personal history of VTE *(N=20)*	2/20 (10.0%)
Family history of VTE	3 (14.3%)
Known thrombophilia [b] *(N=19)*	0
Major bleeding in the past 30 days	1 (4.8%)
**Laboratory Tests**	
D-dimer (Positive)[d] *(N=17)*	17/17 (100.0%)
D-dimer levels >5 x upper limit [d] *(N=17)*	8/17 (47.1%)
D-dimer levels >10 x upper limit [d] *(N=17)*	7/17 (41.2%)
Fibrinogen	372.04±38.02
Fibrinogen < 150	2 (9.5%)
INR	3 (14.3%)
Platelet count (IQR)	114 (51-135)
Platelet count	94.29±9.96
Platelet count < 150,000/ fL	21 (100.0%)

Platelet count < 50/ µL	5 (23.8%)
PF-4 antibody tested	5 (23.8%)
PF-4 antibody above normal limit	5/5 (100.0%)
AST	77.63±29.3
ALT	101.4±43.77
**SARS-CoV2 Status**	
Infected	1 (4.8%)
Not infected	20 (95.2%)
Not tested	0
a: At least two of the following: i) upper and/or lower extremity DVT and/or PE; ii) CVST; iii) Splanchnic vein thrombosis. b: Includes antiphospholipid antibody syndrome, inflammatory bowel disease, factor V Leiden, prothrombin G 20210 mutation c: Laboratory tests were performed in each local institution. SEM: standard error of the mean.	

2
